# Supplementary material for: Favorable association between early initiation of sodium-glucose cotransporter-2 inhibitors and in-hospital prognosis in acute myocardial infarction
Source: PLoS One. 2026 Mar 27;21(3):e0345315. doi: 10.1371/journal.pone.0345315 (PMC13028360; doi:10.1371/journal.pone.0345315)
Supplement: S1 Table — (DOCX) [file pone.0345315.s001.docx]

**S1 Table. Comparison of baseline characteristics between SGLT2i and non-SGLT2i groups.**

|  | **All**  **(N = 394)** | **SGLT2i**  **(N = 94)** | **Non-SGLT2i**  **(N = 300)** | **P-value** |
| --- | --- | --- | --- | --- |
| Age (years) | 66.0 ± 11.7 | 68.0 ± 10.0 | 66.5 ± 11.5 | 0.3 |
| Male gender | 225 (57.1) | 56 (59.6) | 169 (56.3) | 0.5 |
| Smoking | 37 (9.4) | 7 (7.4) | 30 (10.0) | 0.4 |
| Hypertension | 320 (81.2) | 81 (86.2) | 239 (79.7) | 0.1 |
| Diabetes | 120 (30.5) | 27 (28.7) | 93 (31.0) | 0.6 |
| Dyslipidemia | 119 (30.2) | 22 (23.4) | 97 (32.3) | 0.1 |
| Heart failure | 64 (16.2) | 19 (20.2) | 45 (15.0) | 0.2 |
| STEMI | 148 (37.6) | 34 (36.2) | 114 (38.0) | 0.7 |
| Shock | 24 (6.1) | 2 (2.1) | 22 (7.3) | 0.06 |
| Sepsis | 149 (37.8) | 29 (30.9) | 120 (40.0) | 0.1 |
| Creatinine (umol/L) | 91.6 (77.9–114.4) | 85.6 (73.3–102.2) | 93.4 (79.2–117.3) | 0.07 |
| Hemoglobin (g/dL) | 12.7 ± 2.2 | 12.3 ± 2.4 | 13.0 ± 1.8 | 0.8 |
| Elevated troponin T | 367 (93.1) | 88 (93.6) | 279 (93.0) | 0.8 |
| NT-proBNP (pg/mL) | 1975 (698–6164) | 2340 (640–4339) | 1375 (243–4372) | 0.05 |
| Ejection fraction (%) | 48 (39–59) | 46 (39–54) | 49 (40–60) | **0.002** |
| Revascularization | 219 (55.6) | 56 (59.6) | 163 (54.3) | 0.4 |
| Heparin | 253 (64.2) | 60 (63.8) | 193 (64.3) | 0.9 |
| Antiplatelets | 355 (90.1) | 86 (91.5) | 269 (89.7) | 0.6 |
| Statins | 337 (85.5) | 83 (88.3) | 254 (84.7) | 0.4 |
| BB/ACEi/ARB/MRA | 275 (69.8) | 82 (87.2) | 193 (64.3) | **< 0.001** |
| *STEMI: ST elevation myocardial infarction, NT-proBNP: N-terminal pro B-type natriuretic peptide, BB: beta blockers, ACEi: angiotensin–converting enzyme inhibitors, ARB: angiotensin receptor blockers, MRA: mineralocorticoid antagonists, SGLT2i: sodium–glucose cotransporter-2 inhibitors.* | | | | |
