# Supplementary material for: Favorable association between early initiation of sodium-glucose cotransporter-2 inhibitors and in-hospital prognosis in acute myocardial infarction
Source: PLoS One. 2026 Mar 27;21(3):e0345315. doi: 10.1371/journal.pone.0345315 (PMC13028360; doi:10.1371/journal.pone.0345315)
Supplement: S2 Table — (DOCX) [file pone.0345315.s002.docx]

**S2 Table.** **Univariable analysis of clinical and treatment characteristics associated with in-hospital outcomes between non-revascularization and revascularization groups.**

|  | **Non-revascularization** | | **Revascularization** | |
| --- | --- | --- | --- | --- |
|  | **OR**  **(95% CI)** | **P-value** | **OR**  **(95% CI)** | **P-value** |
| Age (years) | 1.02 (0.99–1.06) | 0.2 | 1.08 (1.03–1.13) | **0.001** |
| Male gender | 0.93 (0.44–2.09) | 0.9 | 0.66 (0.27–1.61) | 0.3 |
| Shock | 12.17 (3.39–43.73) | **< 0.001** | 5.25 (1.44–19.15) | **0.01** |
| Sepsis | 3.39 (1.49–7.75) | **0.004** | 4.09 (1.63–10.27) | **0.003** |
| Creatinine (umol/L) | 1.04 (1.01–1.07) | **0.01** | 1.02 (1.00–1.03) | 0.08 |
| Hemoglobin (g/dL) | 0.88 (0.73–1.05) | 0.2 | 0.72 (0.57–0.93) | **0.01** |
| NT-proBNP (log-transformed) | 1.59 (1.19–2.10) | **0.001** | 2.19 (1.51–3.16) | **< 0.001** |
| Ejection fraction (%) | 0.99 (0.96–1.01) | 0.3 | 0.94 (0.91–0.98) | **0.001** |
| Heparin | 0.35 (0.16–0.77) | **0.01** | 0.60 (0.25–1.47) | 0.3 |
| Antiplatelets | 0.11 (0.04–0.28) | **< 0.001** | 0.17 (0.06–0.52) | **0.002** |
| Statins | 0.25 (0.11–0.61) | **0.002** | 0.16 (0.06–0.45) | **< 0.001** |
| BB/ACEi/ARB/MRA | 0.09 (0.04–0.23) | **< 0.001** | 0.06 (0.02–0.19) | **< 0.001** |
| SGLT2i | 0.10 (0.01–0.73) | **0.02** | 0.26 (0.06–1.17) | 0.08 |
| *NT-proBNP: N-terminal pro B-type natriuretic peptide, BB: beta blockers, ACEi: angiotensin–converting enzyme inhibitors, ARB: angiotensin receptor blockers, MRA: mineralocorticoid antagonists, SGLT2i: sodium–glucose cotransporter-2 inhibitors.* | | | | |
