# Supplementary material for: Favorable association between early initiation of sodium-glucose cotransporter-2 inhibitors and in-hospital prognosis in acute myocardial infarction
Source: PLoS One. 2026 Mar 27;21(3):e0345315. doi: 10.1371/journal.pone.0345315 (PMC13028360; doi:10.1371/journal.pone.0345315)
Supplement: S3 Table — (DOCX) [file pone.0345315.s003.docx]

**S3 Table. Association between SGLT2i use and in-hospital mortality across multiple univariable and multivariable logistic regression models.**

|  | **Univariable** | | **Multivariable** | |
| --- | --- | --- | --- | --- |
|  | **OR**  **(95% CI)** | **P-value** | **OR**  **(95% CI)** | **P-value** |
| **Model 1** | | | | |
| Age (years) | 1.05 (1.02–1.08) | **< 0.001** | 1.02 (0.99–1.06) | 0.2 |
| Sepsis | 3.87 (2.10–7.14) | **< 0.001** | 2.00 (0.89–4.51) | 0.1 |
| Hemoglobin (g/dL) | 0.79 (0.69–0.92) | **0.002** | 0.85 (0.72–1.01) | 0.06 |
| Ejection fraction (%) | 0.97 (0.95–0.99) | **0.004** | 0.95 (0.93–0.98) | **0.002** |
| Revascularization | 0.52 (0.29–0.93) | **0.02** | 0.66 (0.30–1.45) | 0.3 |
| Antiplatelets | 0.13 (0.06–0.26) | **< 0.001** | 0.14 (0.05–0.37) | **< 0.001** |
| BB/ACEi/ARB/MRA | 0.08 (0.04–0.15) | **< 0.001** | 0.21 (0.09–0.49) | **< 0.001** |
| SGLT2i | 0.16 (0.05–0.54) | **0.003** | 0.19 (0.04–0.93) | **0.04** |
| **Model 2** | | | | |
| Sepsis | 3.87 (2.10–7.14) | **< 0.001** | 2.20 (1.01–4.78) | **0.046** |
| Hemoglobin (g/dL) | 0.79 (0.69–0.92) | **0.002** | 0.83 (0.70–0.98) | **0.036** |
| NT-proBNP (log-transformed) | 1.85 (1.48–2.31) | **< 0.001** | 1.44 (1.11–1.87) | **0.006** |
| Ejection fraction (%) | 0.97 (0.95–0.99) | **0.004** | 0.97 (0.94–1.00) | 0.05 |
| Revascularization | 0.52 (0.29–0.93) | **0.02** | 0.83 (0.38–1.81) | 0.637 |
| Heparin | 0.44 (0.25–0.79) | **0.006** | 0.78 (0.35–1.71) | 0.534 |
| Statins | 0.20 (0.10–0.38) | **< 0.001** | 0.19 (0.08–0.48) | **< 0.001** |
| SGLT2i | 0.16 (0.05–0.54) | **0.003** | 0.12 (0.03–0.55) | **0.006** |
| **Model 3** | | | | |
| Shock | 8.02 (3.38–19.03) | **< 0.001** | 2.79 (0.92–8.53) | 0.07 |
| Sepsis | 3.87 (2.10–7.13) | **< 0.001** | 2.02 (0.89–4.57) | 0.09 |
| Hemoglobin (g/dL) | 0.79 (0.69–0.92) | **0.002** | 0.83 (0.70–0.98) | **0.03** |
| Ejection fraction (%) | 0.97 (0.95–0.99) | **0.004** | 0.95 (0.93–0.98) | **0.003** |
| Revascularization | 0.52 (0.29–0.93) | **0.02** | 0.59 (0.27–1.31) | 0.2 |
| Antiplatelets | 0.13 (0.06–0.26) | **< 0.001** | 0.14 (0.05–0.38) | **< 0.001** |
| BB/ACEi/ARB/MRA | 0.08 (0.04–0.15) | **< 0.001** | 0.24 (0.10–0.57) | **0.001** |
| SGLT2i | 0.16 (0.05–0.54) | **0.003** | 0.21 (0.04–0.99) | **0.04** |
| *NT-proBNP: N-terminal pro B-type natriuretic peptide, BB: beta blockers, ACEi: angiotensin–converting enzyme inhibitors, ARB: angiotensin receptor blockers, MRA: mineralocorticoid antagonists, SGLT2i: sodium–glucose cotransporter-2 inhibitors.* | | | | |
