# Supplementary material for: Favorable association between early initiation of sodium-glucose cotransporter-2 inhibitors and in-hospital prognosis in acute myocardial infarction
Source: PLoS One. 2026 Mar 27;21(3):e0345315. doi: 10.1371/journal.pone.0345315 (PMC13028360; doi:10.1371/journal.pone.0345315)
Supplement: Attachment — Submitted filename: Response to Reviewers R2.docx [file pone.0345315.s007.docx]

**RESPONSE TO THE EDITOR AND REVIEWERS**

**Additional Editor Comments:**

Thank you for submitting your revised manuscript.

Your work is close to acceptance.

However, I would ask the authors to avoid using the term “effect,” as this is an observational study and therefore cannot establish a causal relationship.
For example, a title such as “Favorable association between early initiation of sodium-glucose cotransporter-2 inhibitors and in-hospital prognosis in acute myocardial infarction” would be preferable.

Please revise the manuscript accordingly and ensure that causal language is avoided throughout the manuscript.

*🡪 We sincerely thank the Academic Editor for this important and constructive comment. In response, we have revised the title to: “Favorable Association Between Early Initiation of Sodium-Glucose Cotransporter-2 Inhibitors and In-Hospital Prognosis in Acute Myocardial Infarction.”*

*We have also carefully reviewed the entire manuscript and replaced causal terminology with association-neutral language where appropriate. The abstract, results, discussion, and conclusion sections were revised to emphasize that this observational study identifies associations rather than causal relationships. We further clarified that the findings are hypothesis-generating and warrant confirmation in prospective randomized trials.*

*All modifications are highlighted in red in the revised manuscript.*

**Reviewer #2:**

I would like to thank the authors for their thorough and thoughtful revision. They have adequately addressed all the reviewers’ comments, substantially strengthening the manuscript, particularly with respect to the multivariable analyses, subgroup analyses, and the expanded mechanistic discussion. The methodology is now clearer, the results are robustly supported, and the discussion is well balanced and clinically relevant.

In its current form, I believe the manuscript is suitable for publication.

*🡪 We sincerely thank the reviewer for the thoughtful evaluation and supportive comments. We greatly appreciate the recognition of the strengthened multivariable analyses, subgroup analyses, and expanded mechanistic discussion.*

*To improve numerical precision and clarity, odds ratios are now reported with two decimal places instead of one. In addition, NT-proBNP values were natural log–transformed for regression analyses to enhance model stability and interpretability. These refinements did not alter the statistical significance or the overall interpretation of the findings.*

*All related changes are highlighted in blue in the revised manuscript.*
