# Supplementary material for: Favorable association between early initiation of sodium-glucose cotransporter-2 inhibitors and in-hospital prognosis in acute myocardial infarction
Source: PLoS One. 2026 Mar 27;21(3):e0345315. doi: 10.1371/journal.pone.0345315 (PMC13028360; doi:10.1371/journal.pone.0345315)
Supplement: Attachment — Submitted filename: Reviewer Comments.docx [file pone.0345315.s004.docx]

### ****Reviewer Comments****

I would like to congratulate the authors for this well-designed and clinically relevant study. Please consider the following revisions:

**Minor Revisions:**
The manuscript would benefit from a more detailed characterization of patients with diabetes. Although prevalence is similar between groups, several key aspects remain unclear. Please report baseline HbA1c levels to assess metabolic control, and, if available, information on diabetes-related complications (nephropathy, neuropathy, retinopathy, macrovascular disease) to better describe disease severity. It would also be useful to clarify whether the myocardial infarction was a first event or a recurrence in patients with diabetes. Given the relevance of SGLT2 inhibitors in type 2 diabetes, I suggest performing subgroup analyses to evaluate whether the association between early SGLT2i initiation and outcomes varies by HbA1c categories, CKD severity, or the presence of diabetes complications.

**Major Revisions:**
Tables 2 and 3 show separate multivariable models, but it is unclear whether a single combined model including both clinical and treatment variables was tested. Because LVEF, sepsis, renal function, and revascularization strongly influence prognosis and treatment selection, a unified model is essential to determine whether early SGLT2i initiation remains independently associated with in-hospital mortality. If not performed, I strongly encourage including such a model—at least as a sensitivity analysis—to minimize residual confounding.

**Discussion:**
The Discussion would benefit from a more comprehensive description of the mechanisms that may explain the early benefits of SGLT2 inhibitors in acute MI. Please expand on hemodynamic effects (osmotic diuresis, natriuresis), improvements in vascular function, renal protection, anti-inflammatory and anti-oxidative actions, and especially metabolic remodeling involving increased ketone-based myocardial fuel utilization. To support this, consider citing key mechanistic reviews (Ferrannini 2014; Verma & McMurray 2018; Zelniker & Braunwald 2018; Packer 2020; Santos-Gallego 2019). Strengthening this section will enhance the mechanistic plausibility of your findings.
